# Supplementary material for: Characterization of protein isoform diversity in human umbilical vein endothelial cells via long-read proteogenomics
Source: RNA Biol. 2022 Dec 1;19(1):1228–43. doi: 10.1080/15476286.2022.2141938 (PMC9721438; doi:10.1080/15476286.2022.2141938)
Supplement: Supplemental Material [file KRNB_A_2141938_SM7696.zip › HUVEC_Manuscript_Supplemental_Information_resubmission.pdf]

Table of Contents:

S-1: Cover sheet
S-2: Supplementary Figure S1. Characterization of the HUVEC full-length transcriptome based on long-read RNA-seq data.
S-3: Supplementary Figure S2: Characterization of novel isoform length as defined through long-read proteogenomics
S-4: Supplementary Table S1. Manually curated list of endothelial-relevant genes
S-5: Supplementary Table S2. Information on the major expressed isoform and APPRIS principal annotation for all and endothelial-related genes
S-6: Supplementary Table S3: Splice factors identified within HUVECs
S-6: Supplementary Figure S3. Derivation of predicted protein isoforms to generate a HUVEC sample-specific database.
S-7: Supplementary Table S4. Databases derived from protein isoform models from the long-read proteogenomics platform
S-8: Supplementary Table S5. MetaMorpheus MS search parameters
S-9: Supplementary Figure S4. Basic pH HPLC fractions for the HUVEC MS data collection.
S-10: Supplementary Figure S5. Comparison of proteomic coverage when using the HUVEC sample-specific versus UniProt protein databases for MS searching.
S-11 Supplementary Figure S6. Plectin (PLEC) gene evidenced by seven unique isoforms.
S-12: Supplementary Table S6: Protein isoforms informed by shared and uniquely mapping peptide identifications.
S-13: Supplementary Table S7: Annotation of novel peptides detected from the sample-specific database

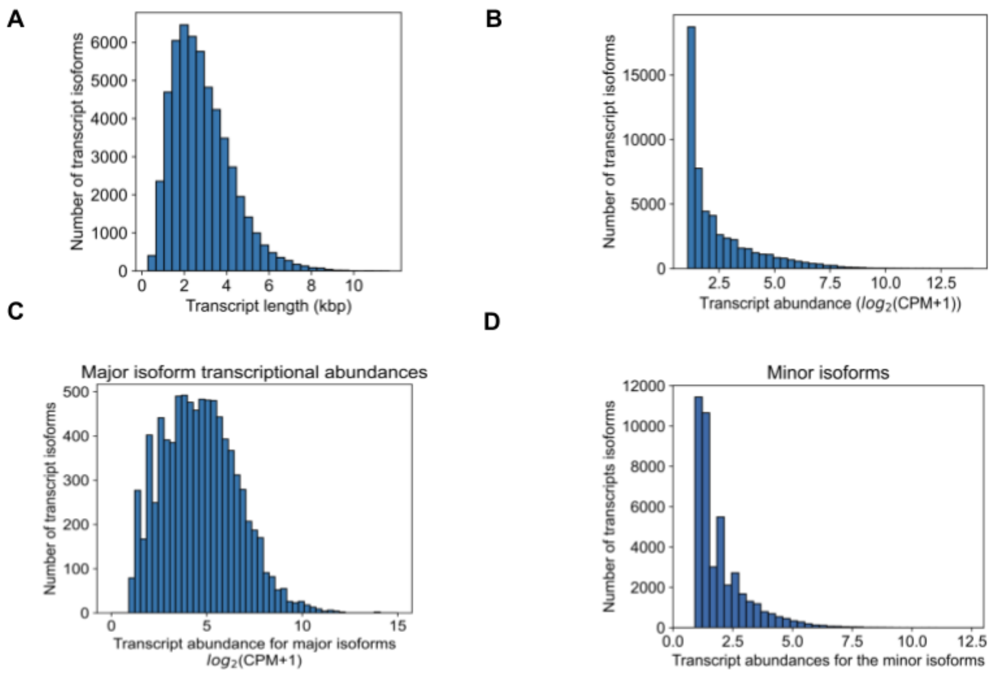

Figure S1. Characterization of the HUVEC full-length transcriptome based on long-read RNA-seq data. A**)** Distribution of transcript isoform length B) Distribution of the transcript abundance (counts per million, CPM) B) Distribution of all transcripts identified from the Iso-Seq pipeline with abundance greater than one CPM. C) Distribution of the abundances foro the most highly expressed isoform for each gene, i.e., the major isoform. D) Distribution of the abundances of the minor isoforms reported for all transcripts with CPM>1.

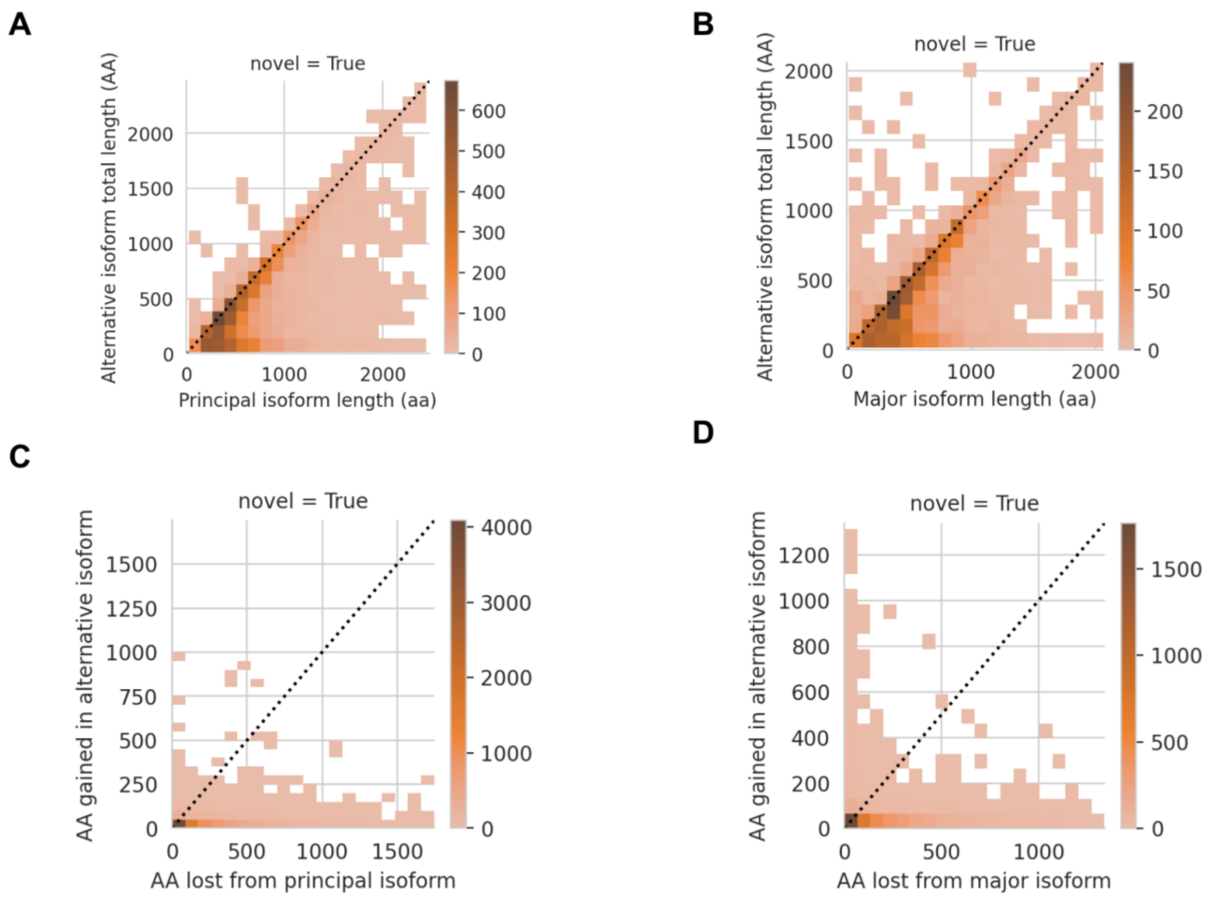

Figure S2. Characterization of novel isoform length as identified through long-read proteogenomics (A) Overall length in amino acids of the principal isoforms as defined by APPRIS (B) Overall length in amino acids of the major isoforms as defined within HUVECs (C) Comparison of the length in amino acids of identified novel isoforms (novel in catalog (NIC) and novel not in catalog (NNC)) against the APPRIS reference isoform. (D) Comparison of the length in amino acids of identified novel isoforms (novel in catalog (NIC) and novel not in catalog (NNC) against the “major isoform” reference isoform as identified within HUVECs.

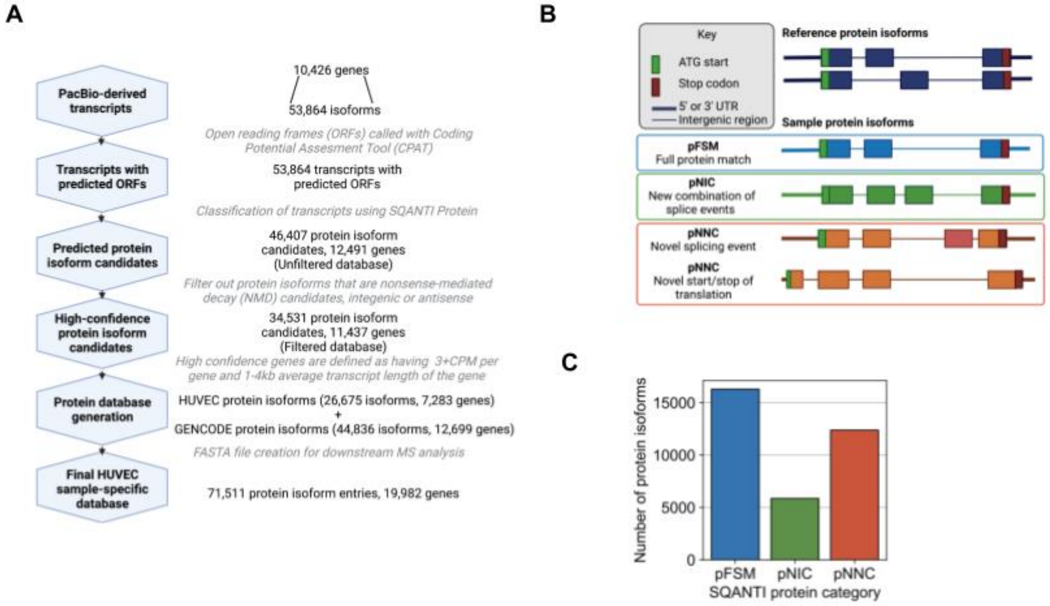

Figure S3. Derivation of predicted protein isoforms to generate a HUVEC sample-specific database.(A) Schematic of protein database generation from long-read RNA-seq data. (B) Schematic of SQANTI Protein classification (C) Bar chart indicating the frequency of different protein isoforms based on novelty category.

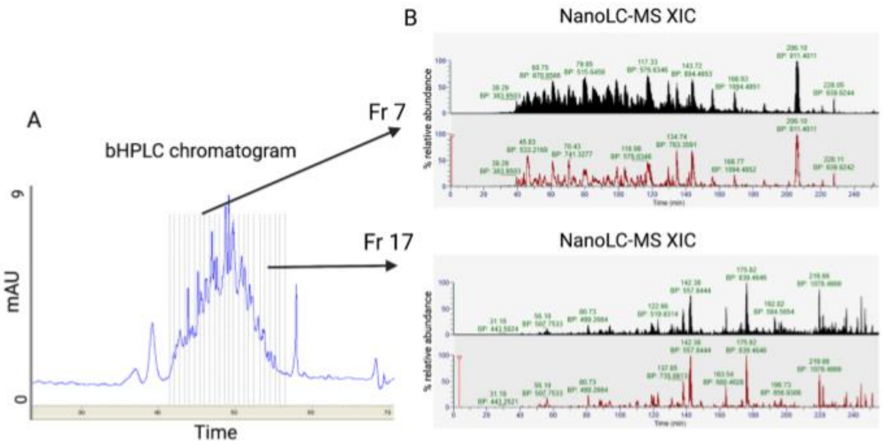

Figure S4. Basic pH HPLC fractions for the HUVEC MS data collection. . (A) UV trace of peptide elution from offline fractionation (range of fractions shown with dashed lines). (B) Total ion (black) and base peak (red) chromatograms from LC-MS analysis of peptides from representative fractions 7 and 17. Basic High Performance Liquid Chromatography (bHPLC), Extracted ion chromatogram (XIC), Nanoscale liquid chromatography coupled to tandem mass spectrometry (nano LC–MS/MS), milli-Absorbance Units (mAU).

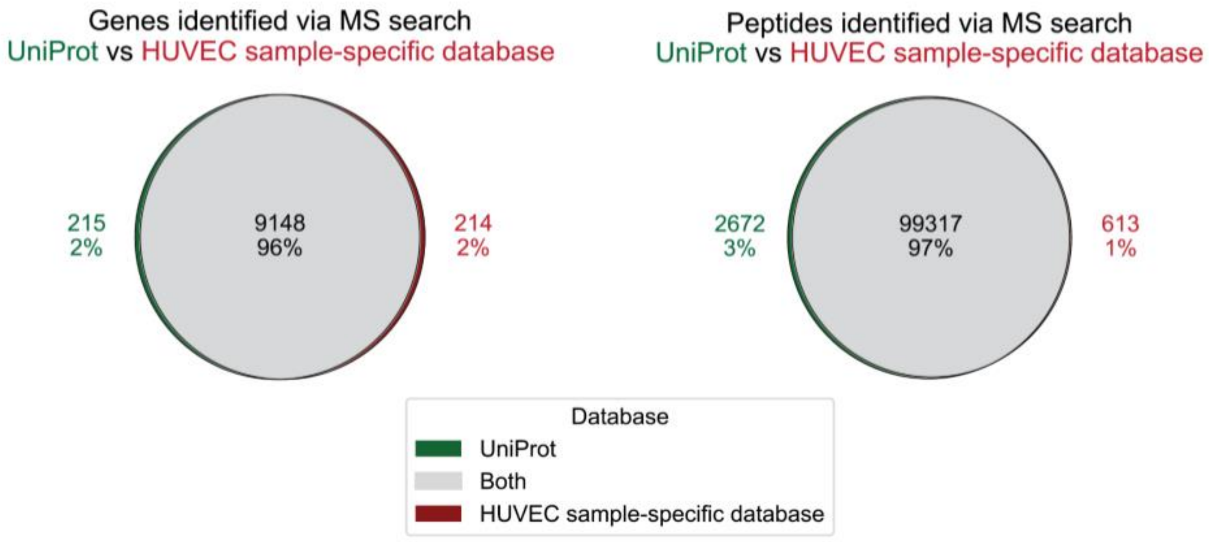

Figure S5. Comparison of proteomic coverage when using the HUVEC sample-specific versus UniProt protein databases for MS searching.

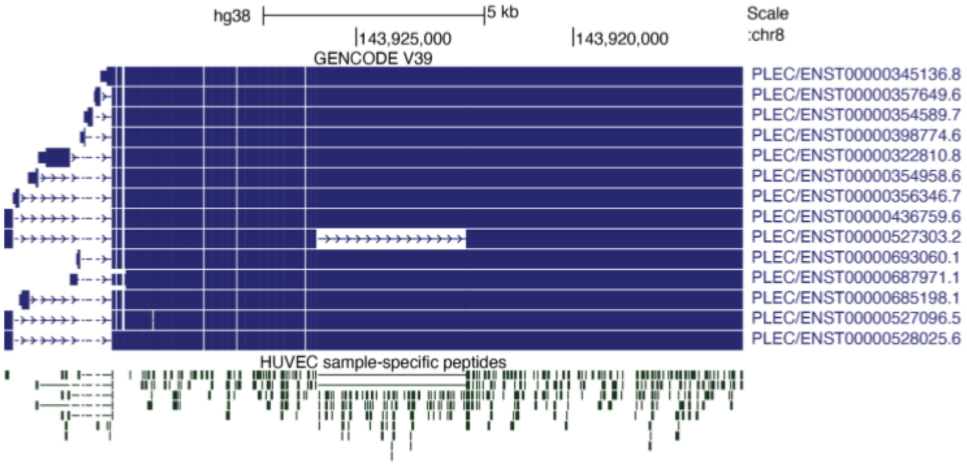

Figure S6. Plectin (*PLEC*) gene evidenced by seven unique isoforms. Plectin (*PLEC*) gene in which MS analysis identified seven unique isoforms each evidenced by their own unique peptide.
