# Supplementary material for: Characterization of protein isoform diversity in human umbilical vein endothelial cells via long-read proteogenomics
Source: RNA Biol. 2022 Dec 1;19(1):1228–43. doi: 10.1080/15476286.2022.2141938 (PMC9721438; doi:10.1080/15476286.2022.2141938)
Supplement: Supplemental Material [file KRNB_A_2141938_SM7696.zip › Supplementary_Information_TableS7.pdf]

Gene	PacBio accession	Peptide sequence	CPM	Raw mass spectrum	Manual spectrum annotation validation notes	Passed Manual Annotation	Raw file name	Scan Number	Annotation of the novelty event	Event of the novel event on the protein isoform sequence	Maps to a single isoform	Link to corresponding UCSC browser track	Isoform track image	Zoom in of image
FXR1	PB.3060.6	GYATDESTVSSVQGSR	6.014723	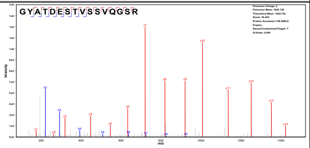	Minimum 9 amino acids, Score>5, delta mass<5ppm, PEP<0.005, good b and y ion series, extra peaks do not exceed 25% above 10% relative intensity; MS2 TIC approx 44% (rough minimum is 20%)	Yes	210827_HUVEC5,6_tryp_HCDorbi_4hr_Fr4	58931	Intron retention event	Insertion	No (PB.3060.6, PB.3060.8)	https://genome.ucsc.edu/cgi-bin/hgTracks?db=hg38&lastVirtModeType=customUrl&lastVirtModeExtraState=multiRegionsBedUrl%3D%2Fuserdata%2Fsessions%2Frr%2F13%2Fmm5db%2F0c7bcefc%2Fhgt%2FcustRgn_genome_1e56d_a22290.bed+0&virtModeType=customUrl&virtMode=1&multiRegionsBedUrl=%2Fuserdata%2Fsessions%2Frr%2F13%2Fmm5db%2F0c7bcefc%2Fhgt%2FcustRgn_genome_1e56d_a22290.bed&nonVirtPosition=chr3%3A180957837%2D180970192&position=multi%3A61641893%2D61642598&hgsid=1323801373_4Vg42e7GvytrAAcReEgOrBP6IHtS	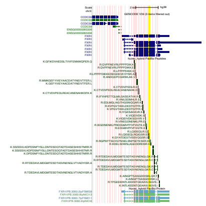	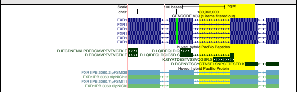
APEH	PB.2610.6	AGPDPGVSPAQVLLSEPEEAAALYR	5.638803	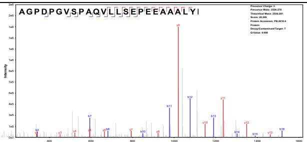	Minimum 9 amino acids, Score>5, delta mass<5ppm, PEP<0.005, good b and y ion series, extra peaks do not exceed 25% above 10% relative intensity; MS2 TIC approx 24% (rough minimum is 20%); y9 ends in proline and is most abundant as expected	Yes	210827_HUVEC5,6_tryp_HCDorbi_4hr_Fr11	220913	Intron retention event	Insertion	No (PB.2610.6, PB.2060.7)	https://genome.ucsc.edu/cgi-bin/hgTracks?db=hg38&lastVirtModeType=customUrl&lastVirtModeExtraState=multiRegionsBedUrl%3D%2Fuserdata%2Fsessions%2Frr%2F13%2Fmm5db%2F0c7bcefc%2Fhgt%2FcustRgn_genome_1e56d_a22290.bed+0&virtModeType=customUrl&virtMode=1&multiRegionsBedUrl=%2Fuserdata%2Fsessions%2Frr%2F13%2Fmm5db%2F0c7bcefc%2Fhgt%2FcustRgn_genome_1e56d_a22290.bed&nonVirtPosition=chr3%3A49674383%2D49674573&position=multi%3A58605299%2D58605489&hgsid=1323801373_4Vg42e7GvytrAAcReEgOrBP6I	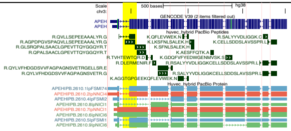	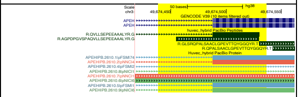
TUBA1A	PB.8359.9	AVFVDLEPTVIGKEDAANNYAR	2.631441	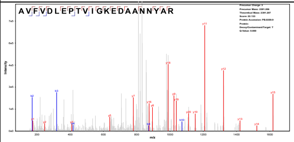	MS2 TIC only 13%, too many missing fragment ions and unassigned peaks, 1 missed cleavage	No	210827_HUVEC5,6_tryp_HCDorbi_4hr_Fr11	148203						
NASP	PB.473.10	ATLVESSTSGFTPGGGGSSVSMR	1.879601	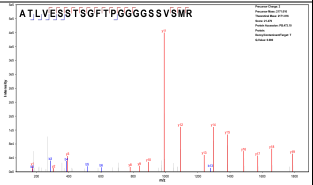	Minimum 9 amino acids, Score>5, delta mass<5ppm, PEP<0.005, good b and y ion series, extra peaks do not exceed 25% above 10% relative intensity; MS2 TIC approx 48% (rough minimum is 20%); y11 ending in proline is most abundant as expected	Yes	210827_HUVEC5,6_tryp_HCDorbi_4hr_Fr12	113107	Exon skipping event	Deletion	Yes (PB.473.10)	https://genome.ucsc.edu/cgi-bin/hgTracks?db=hg38&lastVirtModeType=customUrl&lastVirtModeExtraState=multiRegionsBedUrl%3D%2Fuserdata%2Fsessions%2Frr%2F13%2Fmm5db%2F0c7bcefc%2Fhgt%2FcustRgn_genome_1e56d_a22290.bed+0&virtModeType=customUrl&virtMode=1&multiRegionsBedUrl=%2Fuserdata%2Fsessions%2Frr%2F13%2Fmm5db%2F0c7bcefc%2Fhgt%2FcustRgn_genome_1e56d_a22290.bed&nonVirtPosition=chr	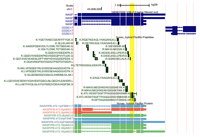	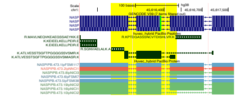
NASP	PB.473.10	ATLVESSTSGFTPGGGGSSVSMR	1.879601	same sequence as above			210827_HUVEC5,6_tryp_HCDorbi_4hr_Fr7	83077						
MAP4	PB.2568.9	KKPCSETSQIEGSPTEFLEEK	3.007362	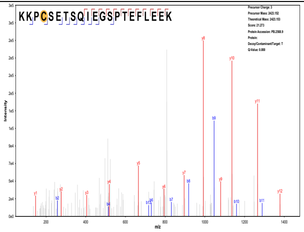	Minimum 9 amino acids, Score>5, delta mass<5ppm, PEP<0.005, good b and y ion series, extra peaks do not exceed 25% above 10% relative intensity; MS2 TIC approx 27% (rough minimum is 20%); y8 ending in proline is most abundant as expected	Yes	210827_HUVEC5,6_tryp_HCDorbi_4hr_Fr8	86426	Exon skipping event	Deletion	Yes (PB.2568.9)	https://genome.ucsc.edu/cgi-bin/hgTracks?db=hg38&lastVirtModeType=customUrl&lastVirtModeExtraState=multiRegionsBedUrl%3D%2Fuserdata%2Fsessions%2Frr%2F13%2Fmm5db%2F0c7bcefc%2Fhgt%2FcustRgn_genome_1e56d_a22290.bed+0&virtModeType=customUrl&virtMode=1&multiRegionsBedUrl=%2Fuserdata%2Fsessions%2Frr%2F13%2Fmm5db%2F0c7bcefc%2Fhgt%2FcustRgn_genome_1e56d_a22290.bed&nonVirtPosition=chr3%3A47928247%2D47998821&position=multi%3A58410922%2D58413847&hgsid=1323801373_4Vg42e7GvytrAAcReEgOrBP6I	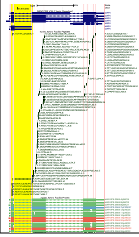	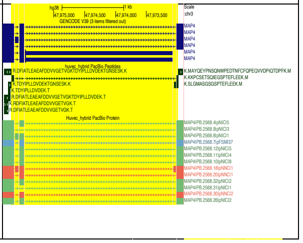

PCBP2	PB.8415.24|PB.8415.27|PB.8415.75	YSTGSDSASFPHTTPSMCLNPDLEGPPLELTK		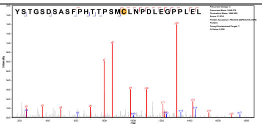	There are many prolines, which might explain why there are so few b ions; meets all other criteria but PEP hovers around minimum criterion	Yes	210827_HUVEC5,6_tryp_HCDorbi_4hr_Fr12	185556	Exon skipping event	Deletion	No(PB.8415.24,PB.8415.27,PB.8415.75)	https://genome.ucsc.edu/cgi-bin/hgTracks?db=hg38&lastVirtModeType=customUrl&lastVirtModeExtraState=multiRegionsBedUrl%3D%2Fuserdata%2Fsessions%2Frr%2F13%2Fmm5db%2F0c7bcefc%2Fhgt%2FcustRgn_genome_1e56d_a22290.bed+0&virtModeType=customUrl&virtMode=1&multiRegionsBedUrl=%2Fuserdata%2Fsessions%2Frr%2F13%2Fmm5db%2F0c7bcefc%2Fhgt%2FcustRgn_genome_1e56d_a22290.bed&nonVirtPosition=chr12%3A53462482%2D53467240&position=multi%3A19540330%2D19540670&hgsid=1323801373_4Vg42e7GvdrAAcReEgOrBP	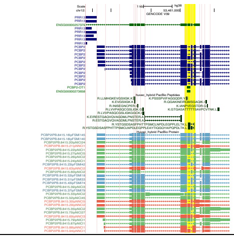	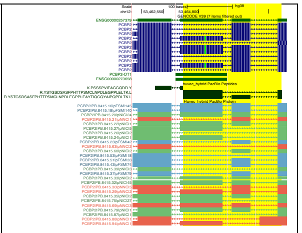
SCAMP3	PB.954.9	DGGNPFAEPSELDNPFQASAAAATAELLK	0.75184	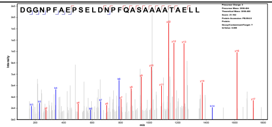	MS2 TIC only 16%, PEP barely meets criterion, too many missing fragment ions and unassigned peaks	No	210827_HUVEC5,6_tryp_HCDorbi_4hr_Fr14	222797						
DAB2IP	PB.6630.12|PB.6630.3|PB.6630.9	MVIENDLSGSSGVQPSPAR		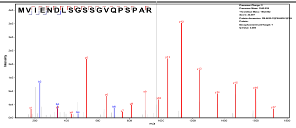	Minimum 9 amino acids, Score>5, delta mass<5ppm, PEP<0.005, good b and y ion series, extra peaks do not exceed 25% above 10% relative intensity; MS2 TIC approx 48% (rough minimum is 20%); y5 ending in proline is	Yes	210827_HUVEC5,6_tryp_HCDorbi_4hr_Fr9	87656	Alternative acceptor (3' splice site)	Deletion	No (PB.6630.3, PB.6630.9, PB.6630.12)	https://genome.ucsc.edu/cgi-bin/hgTracks?db=hg38&lastVirtModeType=customUrl&lastVirtModeExtraState=multiRegionsBedUrl%3D%2Fuserdata%2Fsessions%2Frr%2F13%2Fmm5db%2F0c7bcefc%2Fhgt%2FcustRgn_genome_1e56d_a22290.bed+0&virtModeType=customUrl&virtMode=1&multiRegionsBedUrl=%2Fuserdata%2Fsessions%2Frr%2F1	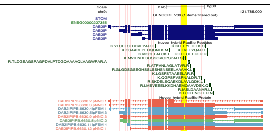	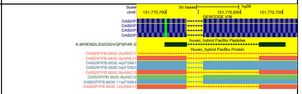
ARVCF	PB.13187.18|PB.13187.2|PB.13187.35|PB.13187.4|PB.13187.44	GASSAGEASEKEPLKGPGPASCS		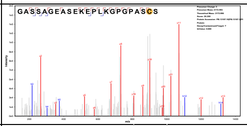	MS2 TIC at 20%, PEP just above minimum, significant unassigned fragment ions, 2 missed cleavages	No	210827_HUVEC5,6_tryp_HCDorbi_4hr_Fr4	42889						
PRKCSH	PB.11912.16	AQQEQELAADAFKELDDDMDGTLLLPRPR	1.127761	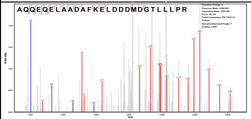	MS2 TIC only 13.5%, PEP just above minimum, significant unassigned fragment ions, 1 missed cleavages	No	210827_HUVEC5,6_tryp_HCDorbi_4hr_Fr10	213920						
UBAP2	PB.6339.16	SQQTLDTTSSVPAPK	3.759202	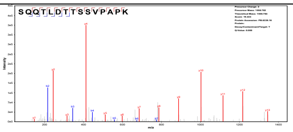	Minimum 9 amino acids, Score>5, delta mass<5ppm, PEP<0.005, good b and y ion series, extra peaks do not exceed 25% above 10% relative intensity; MS2 TIC approx 43% (rough minimum is 20%); y4 ending in proline is among most abundant as expected	Yes	210827_HUVEC5,6_tryp_HCDorbi_4hr_Fr6	52561	Alternative acceptor (3' splice site)	Insertion	No (PB.6339.7, 6339.16)	https://genome.ucsc.edu/cgi-bin/hgTracks?db=hg38&lastVirtModeType=customUrl&lastVirtModeExtraState=multiRegionsBedUrl%3D%2Fuserdata%2Fsessions%2Frr%2F13%2Fmm5db%2F0c7bcefc%2Fhgt%2FcustRgn_genome_1e56d_a22290.bed+0&virtModeType=customUrl&virtMode=1&multiRegionsBedUrl=%2Fuserdata%2Fsessions%2Frr%2F13%2Fmm5db%2F0c7bcefc%2Fhgt%2FcustRgn_genome_1e56d_a22290.bed&nonVirtPosition=chr	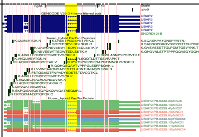	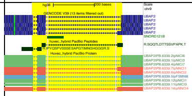
FAM120A	PB.6511.1	APAAAMGVQGFQDYIEK	0.75184	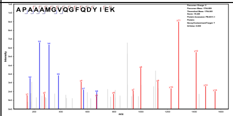	Minimum 9 amino acids, Score>5, delta mass<5ppm, PEP<0.005, good b and y ion series, extra peaks do not exceed 25% above 10% relative intensity; MS2 TIC approx 42% (rough minimum is 20%)	Yes	210827_HUVEC5,6_tryp_HCDorbi_4hr_Fr12	154811	Usages of an ATG start that is further upstream than the ATG start annotated in GENCODE	Insertion	Yes (PB.6511.1)	https://genome.ucsc.edu/cgi-bin/hgTracks?db=hg38&lastVirtModeType=customUrl&lastVirtModeExtraState=multiRegionsBedUrl%3D%2Fuserdata%2Fsessions%2Frr%2F13%2Fmm5db%2F0c7bcefc%2Fhgt%2FcustRgn_genome_1e56d_a22290.bed+0&virtModeType=customUrl&virtMode=1&multiRegionsBedUrl=%2Fuserdata%2Fsessions%2Frr%2F1	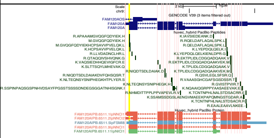	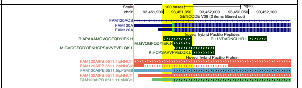
FAM120A	PB.6511.1	APAAAMGVQGFQDYIEK	0.75184	same sequence as above (oxidized methionine version of a peptide already listed earlier in the table)	Doesn't meet PEP score criterion (since this sequence is just the Mox version of the peptide above, I don't think it makes sense to track down the other spectra)		210827_HUVEC5,6_tryp_HCDorbi_4hr_Fr12	126162						

MAP4	PB.2568.11	MYHDDDLADLVFPSSATADTSIFAGQNDPLKDSYVPLELAK	3.759202	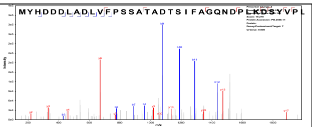	Meets criteria but my only concern is that the y and b ions are not complementary and fairly sparse. This is likely because it's a z=4 ion and those don't tend to fragment very well. Would like to look at the raw data	Yes	210827_HUVEC5,6_tryp_HCDorbi_4hr_Fr13	242854	Exon skipping event	Deletion	Yes (PB.2568.11)	https://genome.ucsc.edu/cgi-bin/hgTracks?db=hg38&lastVirtModeType=customUrl&lastVirtModeExtraState=multiRegionsBedUrl%3D%2Fuserdata%2Fsessions%2Frr%2F13%2Fmm5db%2F0c7bcefc%2Fhgt%2FcustRgn_genome_1e56d_a22290.bed+0&virtModeType=customUrl&virtMode=1&multiRegionsBedUrl=%2Fuserdata%2Fsessions%2Frr%2F13%2Fmm5db%2F0c7bcefc%2Fhgt%2FcustRgn_genome_1e56d_a22290.bed&nonVirtPosition=chr3%3A47916970%2D47928353&position=multi%3A58410401%2D58411028&hgsid=1323801373_4Vg42e7GvvtrAAcReFqOrBP6I	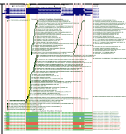	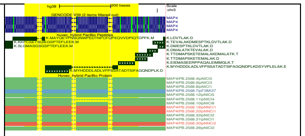
MAP4	PB.2568.11	MYHDDDLADLVFPSSATADTSIFAGQNDPLKDSYVPLELAK	3.759202	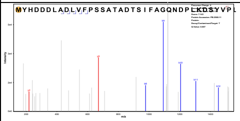	Too few y and b ions, PEP doesn't meet criterion, too many unassigned fragment ions	No	210827_HUVEC5,6_tryp_HCDorbi_4hr_Fr13	241977						
ACTN4	PB.12220.36	AGTQIAAIAQELNELDYYDSHNVNTR	2.631441	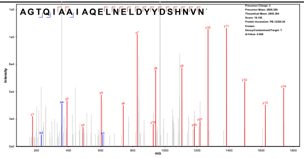	Y ion series looks very nice but the delta mass is over 300ppm, isolation of the C13 isotope	Yes	210827_HUVEC5,6_tryp_HCDorbi_4hr_Fr13	217945	Exon skipping event	Deletion	No (PB.12220.10,		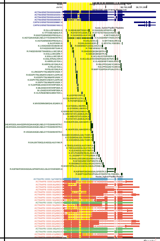	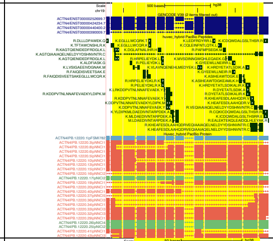
PGAM5	PB.8829.1	EGAPIEPDPPVSHWKPEAVYYEDGAR	22.55521	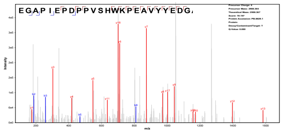	Minimum 9 amino acids, Score>5, delta mass<5ppm, PEP<0.005, good y ion series; a couple of complementary peaks, extra peaks may exceed 25% above 10% relative intensity; MS2 TIC only 16% (rough minimum is 20%); fragmentation actually looks decent considering it is a z=4	Yes	210827_HUVEC5,6_tryp_HCDorbi_4hr_Fr8	126553	Alternative acceptor (3' splice site)	Deletion	Yes(PB.8829.1)	https://genome.ucsc.edu/cgi-bin/hgTracks?db=hg38&lastVirtModeType=customUrl&lastVirtModeExtraState=multiRegionsBedUrl%3D%2Fuserdata%2Fsessions%2Frr%2F13%2Fmm5db%2F0c7bcefc%2Fhgt%2FcustRgn_genome_1e56d_a22290.bed+0&virtModeType=customUrl&virtMode=1&multiRegionsBedUrl=%2Fuserdata%2Fsessions%2Frr%2F13%2Fmm5db%2F0c7bcefc%2Fhgt%2FcustRgn_genome_1e56d_a22290.bed&nonVirtPosition=chr12%3A132717729%2D1327180	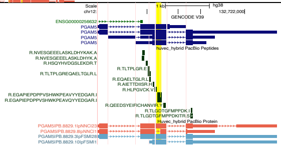	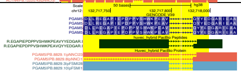
ARRB1	PB.7883.9	TASSDDDIVFEDFAR	0.75184	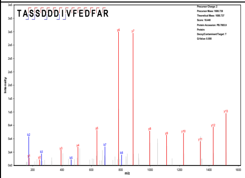	Minimum 9 amino acids, Score>5, delta mass<5ppm, PEP<0.005, good b and y ion series, extra peaks do not exceed 25% above 10% relative intensity; MS2 TIC approx 44% (rough minimum is 20%)	Yes	210827_HUVEC5,6_tryp_HCDorbi_4hr_Fr4	171067	Novel exon	Insertion	Yes (PB.7883.9)	https://genome.ucsc.edu/cgi-bin/hgTracks?db=hg38&lastVirtModeType=customUrl&lastVirtModeExtraState=multiRegionsBedUrl%3D%2Fuserdata%2Fsessions%2Frr%2F13%2Fmm5db%2F0c7bcefc%2Fhgt%2FcustRgn_genome_1e56d_a22290.bed+0&virtModeType=customUrl&virtMode=1&multiRegionsBedUrl=%2Fuserdata%2Fsessions%2Frr%2F13%2Fmm5db%2F0c7bcefc%2Fhgt%2FcustRgn_genome_1e56d_a22290.bed&nonVirtPosition=chr	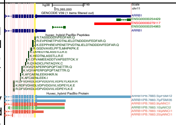	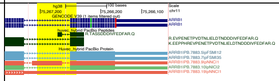
SCAMP3	PB.954.9	DGGNPFAEPSELDNPFQASAAAATAELLK	0.75184	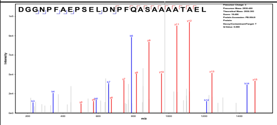	Minimum 9 amino acids, Score>5, delta mass<5ppm, PEP<0.005, good b and y ion series, extra peaks do not exceed 25% above 10% relative intensity; MS2 TIC approx 41% (rough minimum is 20%)	Yes	210827_HUVEC5,6_tryp_HCDorbi_4hr_Fr13	249752	Exon skipping event	Deletion	Yes (PB.954.9)	https://genome.ucsc.edu/cgi-bin/hgTracks?db=hg38&lastVirtModeType=customUrl&lastVirtModeExtraState=multiRegionsBedUrl%3D%2Fuserdata%2Fsessions%2Frr%2F13%2Fmm5db%2F0c7bcefc%2Fhgt%2FcustRgn_genome_1e56d_a22290.bed+0&virtModeType=customUrl&virtMode=1&multiRegionsBedUrl=%2Fuserdata%2Fsessions%2Frr%2F13%2Fmm5db%2F0c7bcefc%2Fhgt%2FcustRgn_genome_1e56d_a22290.bed&nonVirtPosition=chr1%3A155260421%2D15526218	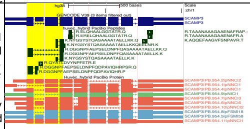	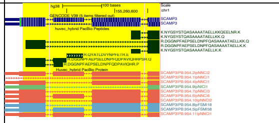

EDC4	PB.10482.10|PB.10482.9	STRPAPGPFLSYGAER		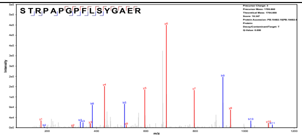	Looks really good except for mass difference (>500ppm), isolation of the C13 isotope	Yes	210827_HUVEC5,6_tryp_HCDorbi_4hr_Fr15	72346	Intron retention event	Insertion	No (PB.10482.8, PB.10482.9)	https://genome.ucsc.edu/cgi-bin/hgTracks?db=hg38&lastVirtModeType=customUrl&lastVirtModeExtraState=multiRegionsBedUrl%3D%2Fuserdata%2Fsessions%2Frr%2F13%2Fmm5db%2F0c7bcefc%2Fhgt%2FcustRgn_genome_1e56d_a22290.bed+0&virtModeType=customUrl&virtMode=1&multiRegionsBedUrl=%2Fuserdata%2Fsessions%2Frr%2F13%2Fmm5db%2F0c7bcefc%2Fhgt%2FcustRgn_genome_1e56d_a22290.bed&nonVirtPosition=chr16%3A67873052%2D67884499&position=multi%3A33125092%2D33130630&hgsid=133133675 [illegible]	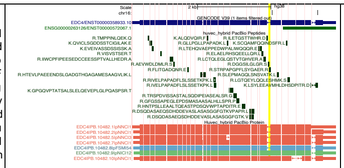	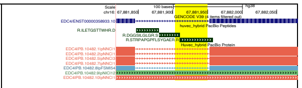
RBMS1	PB.2016.3	MIFPSSSGNPGGSSNCR	47.74187	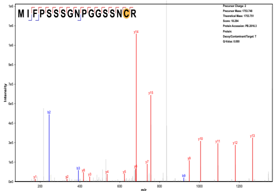	Minimum 9 amino acids, Score>5, delta mass<5ppm, PEP<0.005, good b and y ion series, extra peaks do not exceed 25% above 10% relative intensity; MS2 TIC approx 29% (rough minimum is 20%); y14 ending in proline is most abundant	Yes	210827_HUVEC5,6_tryp_HCDorbi_4hr_Fr15	61807	Alternative first exon	Substitution	Yes (PB.2016.3)	https://genome.ucsc.edu/cgi-bin/hgTracks?db=hg38&lastVirtModeType=customUrl&lastVirtModeExtraState=multiRegionsBedUrl%3D%2Fuserdata%2Fsessions%2Frr%2F13%2Fmm5db%2F0c7bcefc%2Fhgt%2FcustRgn_genome_1e56d_a22290.bed+0&virtModeType=customUrl&virtMode=1&multiRegionsBedUrl=%2Fuserdata%2Fsessions%2Frr%2F13%2Fmm5db%2F0c7bcefc%2Fhgt%2FcustRgn_genome_1e56d_a22290.bed&nonVirtPosition=chr [illegible]	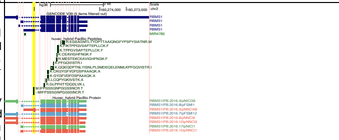	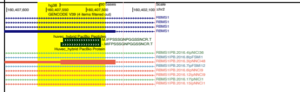
RBMS1	PB.2016.3	MIFPSSSGNPGGSSNCR	47.74187	same sequence as above (oxidized methionine version of a peptide already listed earlier in the table)	Minimum 9 amino acids, Score>5, delta mass<5ppm, PEP<0.005, good b and y ion series, extra peaks do not exceed 25% above 10% relative intensity; MS2 TIC approx 29% (rough minimum is 20%); y8 ending in proline is most abundant		210827_HUVEC5,6_tryp_HCDorbi_4hr_Fr15	49330				https://genome.ucsc.edu/cgi-bin/hgTracks?db=hg38&lastVirtModeType=customUrl&lastVirtModeExtraState=multiRegionsBedUrl%3D%2Fuserdata%2Fsessions%2Frr%2F13%2Fmm5db%2F0c7bcefc%2Fhgt%2FcustRgn_genome_1e56d_a22290.bed+0&virtModeType=customUrl&virtMode=1&multiRegionsBedUrl=%2Fuserdata%2Fsessions%2Frr%2F13%2Fmm5db%2F0c7bcefc%2Fhgt%2FcustRgn_genome_1e56d_ [illegible]	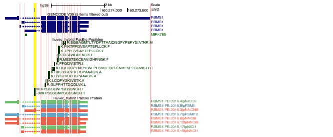	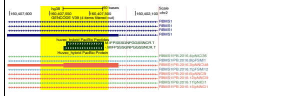
S100PBP	PB.335.4	SLVVSTSSNKDVLNKDSGK	14.66089	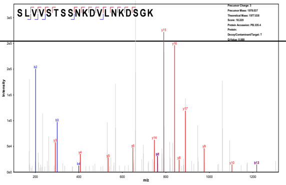	Mass difference too high (>500ppm), isolation of the C13 isotope	Yes	210827_HUVEC5,6_tryp_HCDorbi_4hr_Fr11	43051	Alternative acceptor (3' splice site)	Deletion	Yes (PB.335.4)	https://genome.ucsc.edu/cgi-bin/hgTracks?db=hg38&lastVirtModeType=customUrl&lastVirtModeExtraState=multiRegionsBedUrl%3D%2Fuserdata%2Fsessions%2Frr%2F13%2Fmm5db%2F0c7bcefc%2Fhgt%2FcustRgn_genome_1e56d_a22290.bed+0&virtModeType=customUrl&virtMode=1&multiRegionsBedUrl=%2Fuserdata%2Fsessions%2Frr%2F1 [illegible]	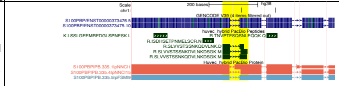	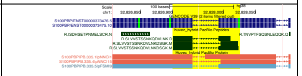
RPA1	PB.10683.5	IGNPVPYNEGSTVSK	1.503681	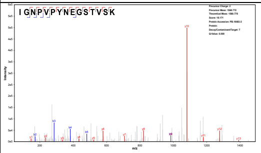	Minimum 9 amino acids, Score>5, delta mass<5ppm, PEP<0.005, good b and y ion series, extra peaks MAY exceed 25% above 10% relative intensity; MS2 TIC approx 17% (rough minimum is 20%); y10 ending in proline is most abundant	Yes	210827_HUVEC5,6_tryp_HCDorbi_4hr_Fr8	66226	Exon skipping event	Deletion	Yes (PB.10683.5)	https://genome.ucsc.edu/cgi-bin/hgTracks?db=hg38&lastVirtModeType=customUrl&lastVirtModeExtraState=multiRegionsBedUrl%3D%2Fuserdata%2Fsessions%2Frr%2F13%2Fmm5db%2F0c7bcefc%2Fhgt%2FcustRgn_genome_1e56d_a22290.bed+0&virtModeType=customUrl&virtMode=1&multiRegionsBedUrl=%2Fuserdata%2Fsessions%2Frr%2F1 [illegible]	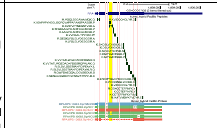	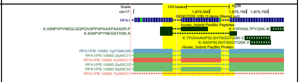
PECAM1	PB.11293.45|PB.11293.80	ELELLTSKDPPPSASQSAGITDLGKK		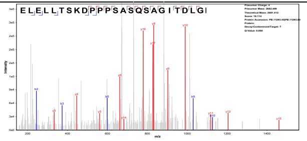	Mass difference too high (>300ppm), isolation of the C13 isotope	Yes	210827_HUVEC5,6_tryp_HCDorbi_4hr_Fr11	112520	Novel exon	Insertion	No (PB.11293.45, PB.11293.80)	https://genome.ucsc.edu/cgi-bin/hgTracks?db=hg38&lastVirtModeType=customUrl&lastVirtModeExtraState=multiRegionsBedUrl%3D%2Fuserdata%2Fsessions%2Frr%2F13%2Fmm5db%2F0c7bcefc%2Fhgt%2FcustRgn_genome_1e56d_a22290.bed+0&virtModeType=customUrl&virtMode=1&multiRegionsBedUrl=%2Fuserdata%2Fsessions%2Frr%2F13%2Fmm5db%2F0c7bcefc%2Fhgt%2FcustRgn_genome_1e56d_a22290.bed&nonVirtPosition=chr17%3A64319415%2D64390860&position=multi%3A37911920%2D37919452&hgsid=133133675 [illegible]	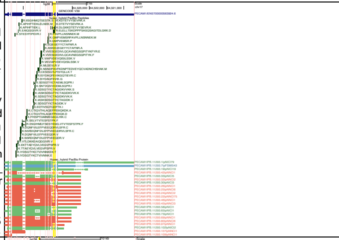	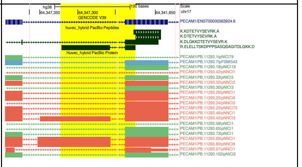
NHSL1	PB.4914.1	CAGDIFGELEGQAAALGHR	1.879601	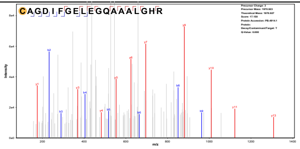	Minimum 9 amino acids, Score>5, delta mass<5ppm, PEP<0.005, good b and y ion series, extra peaks exceed 25% above 10% relative intensity; MS2 TIC approx 15% (rough minimum is 20%)	Yes	210827_HUVEC5,6_tryp_HCDorbi_4hr_Fr12	195591	Alternative first exon	Substitution	Yes	https://genome.ucsc.edu/cgi-bin/hgTracks?db=hg38&lastVirtModeType=customUrl&lastVirtModeExtraState=multiRegionsBedUrl%3D%2Fuserdata%2Fsessions%2Frr%2F13%2Fmm5db%2F0c7bcefc%2Fhgt%2FcustRgn_genome_1e56d_a22290.bed+0&virtModeType=customUrl&virtMode=1&multiRegionsBedUrl=%2Fuserdata%2Fsessions%2Frr%2F13%2Fmm5db%2F0c7bcefc%2Fhgt%2FcustRgn_genome_1e56d_a22290.bed&nonVirtPosition=chr6%3A138414829%2D138903008&position=multi%3A73908187%2D73929828&hgsid=1323801 [illegible]	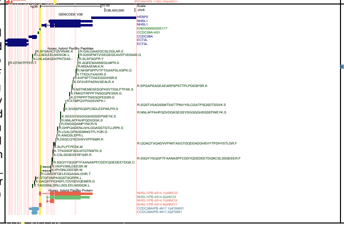	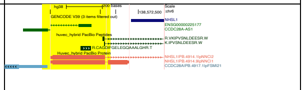

TGM2	PB.12826.47	VVNCNDDQGVLLGR	14.28497	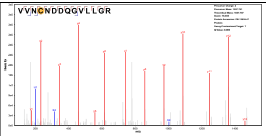	Minimum 9 amino acids, Score>5, delta mass<5ppm, PEP<0.005, good b and y ion series, extra peaks do not exceed 25% above 10% relative intensity; MS2 TIC approx 45% (rough minimum is 20%)	Yes	210827_HUVEC5,6_tryp_HCDorbi_4hr_Fr9	69178	Alternative donor (5' splice site)	Deletion	Yes	https://genome.ucsc.edu/cgi-bin/hgTracks?db=hg38&lastVirtModeType=customUrl&lastVirtModeExtraState=multiRegionsBedUrl%3D%2Fuserdata%2Fsessions%2Frr%2F13%2Fmm5db%2F0c7bcefc%2Fhgt%2FcustRgn_genome_1e56d_a22290.bed+0&virtModeType=customUrl&virtMode=1&multiRegionsBedUrl=%2Fuserdata%2Fsessions%2Frr%2F13%2Fmm5db%2F0c7bcefc%2Fhgt%2FcustRgn_genome_1e56d_a22290.bed&nonVirtPosition=chr20%3A38127385%2D38165270&position=multi%3A53303427%2D53308980&hgsid=1323801373_4Vq42e7GvytrAAcReEqOrBP	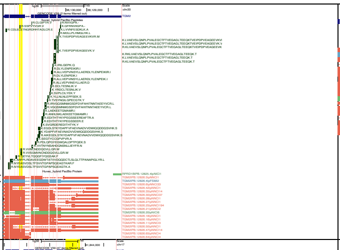	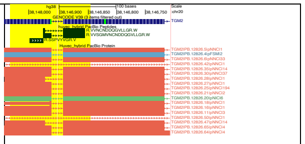
P4HB	PB.11450.14	MDSTANEVIDYNGER	9.022085	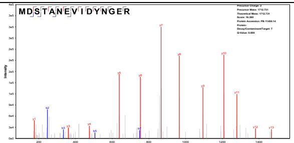	Minimum 9 amino acids, Score>5, delta mass<5ppm, PEP<0.005, good b and y ion series, extra peaks do not exceed 25% above 10% relative intensity; MS2 TIC approx 38% (rough minimum is 20%)	Yes	210827_HUVEC5,6_tryp_HCDorbi_4hr_Fr4	98410	Alternative donor (5' splice site)	Deletion	Yes	https://genome.ucsc.edu/cgi-bin/hgTracks?db=hg38&lastVirtModeType=customUrl&lastVirtModeExtraState=multiRegionsBedUrl%3D%2Fuserdata%2Fsessions%2Frr%2F13%2Fmm5db%2F0c7bcefc%2Fhgt%2FcustRgn_genome_1e56d_a22290.bed+0&virtModeType=customUrl&virtMode=1&multiRegionsBedUrl=%2Fuserdata%2Fsessions%2Frr%2F13%2Fmm5db%2F0c7bcefc%2Fhgt%2FcustRgn_genome_1e56d_a22290.bed&nonVirtPosition=chr17%3A81843166%2D81860535&position=multi%3A38835608%2D38838689&hgsid=1323801373_4Vq42e7GvytrAAcReEqOrBP	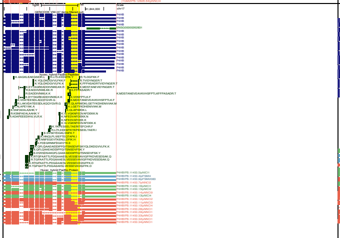	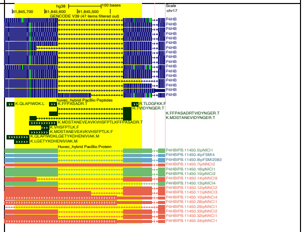
RBMS1	PB.2016.3	IFPSSSGNPGGSSNCR	47.74187	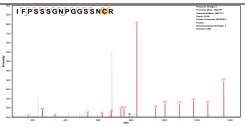	Minimum 9 amino acids, Score>5, delta mass<5ppm, PEP<0.005, good b and y ion series, extra peaks do not exceed 25% above 10% relative intensity; MS2 TIC approx 34% (rough minimum is 20%)	Yes	210827_HUVEC5,6_tryp_HCDorbi_4hr_Fr14	38909	Alternative first exon	Insertion	Yes (PB.2016.3)	https://genome.ucsc.edu/cgi-bin/hgTracks?db=hg38&lastVirtModeType=customUrl&lastVirtModeExtraState=multiRegionsBedUrl%3D%2Fuserdata%2Fsessions%2Frr%2F13%2Fmm5db%2F0c7bcefc%2Fhgt%2FcustRgn_genome_1e56d_a22290.bed+0&virtModeType=customUrl&virtMode=1&multiRegionsBedUrl=%2Fu	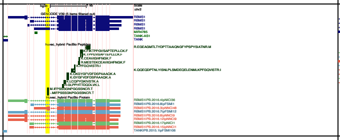	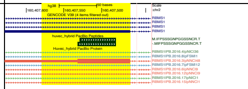
RBMS1	PB.2016.3	MIFPSSSGNPGGSSNCR	47.74187	same sequence as above (oxidized methionine version of a peptide already listed earlier in the table)	Minimum 9 amino acids, Score>5, delta mass<5ppm, PEP<0.005, good b and y ion series, extra peaks do not exceed 25% above 10% relative intensity; MS2 TIC approx 29% (rough minimum is 20%)		210827_HUVEC5,6_tryp_HCDorbi_4hr_Fr15	61807		Insertion	Yes (PB.2016.3)	https://genome.ucsc.edu/cgi-bin/hgTracks?db=hg38&lastVirtModeType=customUrl&lastVirtModeExtraState=multiRegionsBedUrl%3D%2Fuserdata%2Fsessions%2Frr%2F13%2Fmm5db%2F0c7bcefc%2Fhgt%2FcustRgn_genome_1e56d_a22290.bed+0&virtModeType=customUrl&virtMode=1&multiRegionsBedUrl=%2Fu		
RBMS1	PB.2016.3	MIFPSSSGNPGGSSNCR	47.74187	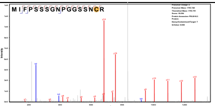	Minimum 9 amino acids, Score>5, delta mass<5ppm, PEP<0.005, good b and y ion series, extra peaks do not exceed 25% above 10% relative intensity; MS2 TIC	Yes	210827_HUVEC5,6_tryp_HCDorbi_4hr_Fr15	49330	Alternative first exon	Insertion	Yes	https://genome.ucsc.edu/cgi-bin/hgTracks?db=hg38&lastVirtModeType=customUrl&lastVirtModeExtraState=multiRegionsBedUrl%3D%2Fuserdata%2Fsessions%2Frr%2F13%2Fmm5db%2F0c7bcefc%2Fhgt%2FcustRgn_genome_1e56d_a22290.bed+0&v	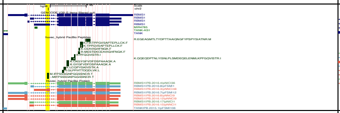	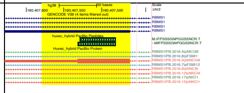
PECAM1	PB.11293.25	SDSGTYICTAEMLSQPR	6.390643	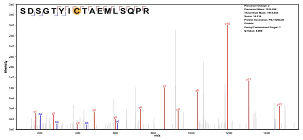	Doesn't meet PEP score criterion; mass difference is close to the 5ppm threshold, lots of unassigned fragment ions	No	210827_HUVEC5,6_tryp_HCDorbi_4hr_Fr10	129601						
SP100	PB.2288.24	AEPTGPEAELHNHGIQINSCSVR	13.53313	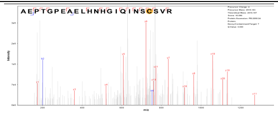	MS2 TIC is only 9.6%; lots of extra peaks exceeding general threshold of 25% greater than 10% relative abundance	No	210827_HUVEC5,6_tryp_HCDorbi_4hr_Fr9	82599						
FTSJ1	PB.13630.7	VDTFPQPLAAPQCHTLLAPEVWK	1.127761	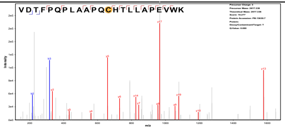	Minimum 9 amino acids, Score>5, delta mass<5ppm, PEP<0.005, good b and y ion series, extra peaks MAY exceed 25% above 10% relative intensity; MS2 TIC approx 28% (rough minimum is 20%)	Yes	210827_HUVEC5,6_tryp_HCDorbi_4hr_Fr16	159097	Intron retention event	Insertion	No (PB.13630.3, PB.13630.7)	https://genome.ucsc.edu/cgi-bin/hgTracks?db=hg38&lastVirtModeType=customUrl&lastVirtModeExtraState=multiRegionsBedUrl%3D%2Fuserdata%2Fsessions%2Frr%2F13%2Fmm5db%2F0c7bcefc%2Fhgt%2FcustRgn_genome_1e56d_a22290.bed+0&virtModeType=customUrl&virtMode=1&multiRegionsBedUrl=%2Fuserdata%2Fsessions%2Frr%2F13%2Fmm5db%2F0c7bcefc%2Fh	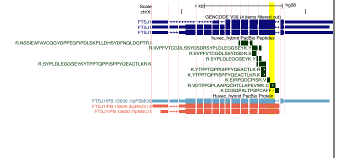	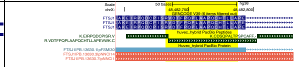

BAG6	PB.4517.86	DLSAMEPNDS TSTAVEEPDS LEVLVK	1.503681	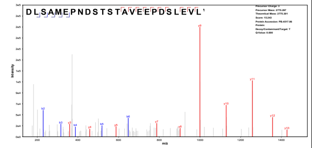	Minimum 9 amino acids, Score>5, delta mass<5ppm, PEP<0.005, good b and y ion series, extra peaks MAY exceed 25% above 10% relative intensity; MS2 TIC approx 24% (rough minimum is 20%)	Yes	210827_HUVEC5,6_ tryp_HCDorbi_4hr_F r6	209310	Alternative first exon	Insertion	Yes (PB.4517.86)	https://genome.ucsc.edu/cgi-bin/hgTracks?db=hg38&lastVirtModeType=customUrl&lastVirtModeExtraState=multiRegionsBedUrl%3D%2Fuserdata%2Fsessions%2Frr%2F13%2Fmm5db%2F0c7bcefc%2Fhgt%2FcustRgn_genome_1e56d_a22290.bed+0&virtModeType=customUrl&virtMode=1&multiRegionsBedUrl=%2Fuserdata%2Fsessions%2Frr%2F13%2Fmm5db%2F0c7bcefc%2Fhgt%2FcustRgn_genome_1e56d_a22290.bed&nonVirtPosition=chr6%3A31639028%2D31652705&	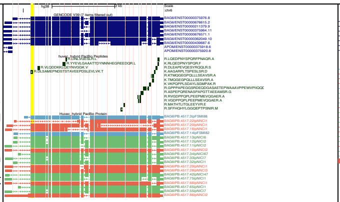	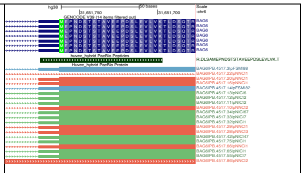
EGFL7	PB.6795.3	GTACLQTVHS VCPR	0.75184	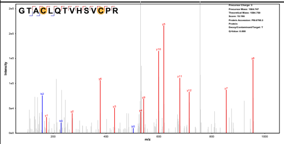	Minimum 9 amino acids, Score>5, delta mass<5ppm, PEP<0.005, good b and y ion series, extra peaks MAY exceed 25% above 10% relative intensity; MS2 TIC approx 28% (rough minimum is 20%)	Yes	210827_HUVEC5,6_ tryp_HCDorbi_4hr_F r12	41752	Frameshift	Different frame of translation leading to portion of the protein with different sequence.	Yes (PB.6795.3)	https://genome.ucsc.edu/cgi-bin/hgTracks?db=hg38&lastVirtModeType=customUrl&lastVirtModeExtraState=multiRegionsBedUrl%3D%2Fuserdata%2Fsessions%2Frr%2F13%2Fmm5db%2F0c7bcefc%2Fhgt%2FcustRgn_genome_1e56d_a22290.bed+0&virtModeType=customUrl&virtMode=1&multiRegionsBedUrl=%2Fuserdata%2Fsessions%2Frr%2F13%2Fmm5db%2F0c7bcefc%2Fh	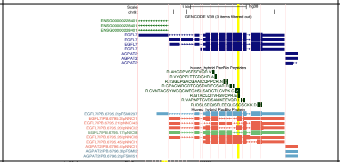	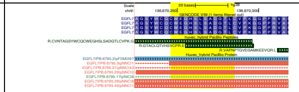
SERPINB6	PB.4349.6	GNTAAQMAQ TESHSVAR	1.879601	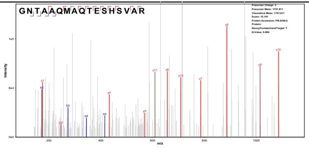	Minimum 9 amino acids, Score>5, delta mass<5ppm, PEP<0.005, good b and y ion series, extra peaks exceed 25% above 10% relative intensity; MS2 TIC approx 10% (rough minimum is 20%)	Yes	210827_HUVEC5,6_ tryp_HCDorbi_4hr_F r6	48710	Novel exon	Insertion	Yes	https://genome.ucsc.edu/cgi-bin/hgTracks?db=hg38&lastVirtModeType=customUrl&lastVirtModeExtraState=multiRegionsBedUrl%3D%2Fuserdata%2Fsessions%2Frr%2F13%2Fmm5db%2F0c7bcefc%2Fhgt%2FcustRgn_genome_1e56d_a22290.bed+0&virtModeType=customUrl&virtMode=1&multiRegionsBedUrl=%2Fuserdata%2Fsessions%2Frr%2F13%2Fmm5db%2F0c7bcefc%2Fhgt%2FcustRgn_genome_1e56d_a22290.bed&nonVirtPosition=chr6%3A2956008%2D2959291&position=multi%3A70289125%2D70289471&hgsid=1329713113_V	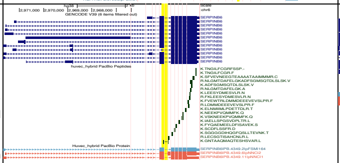	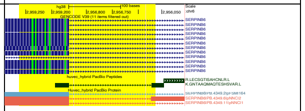
CARNMT1	PB.6454.1	RLEEGQAAAT TLSVAER	0.75184	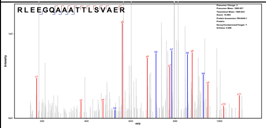	Missed cleavage; extra peaks exceed 25% above 10% relative intensity; MS2 TIC only 8.5%	No	210827_HUVEC5,6_ tryp_HCDorbi_4hr_F r8	78715						
TGFB1I1	PB.10368.2	GLCGSCNKPI AGQVSGAL	5.638803	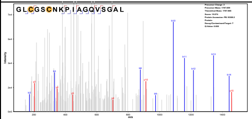	extra peaks exceed 25% above 10% relative intensity; MS2 TIC only 7.4%	No	210827_HUVEC5,6_ tryp_HCDorbi_4hr_F r12	95880						
DPP3	PB.7781.8	GAFNFDQETV INPETGEQVR	10.90169	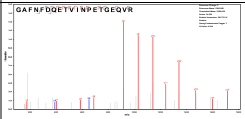	Minimum 9 amino acids, Score>5, delta mass<5ppm, PEP<0.005, good b and y ion series, extra peaks do not exceed 25% above 10% relative intensity; MS2 TIC approx 53% (rough	Yes	210827_HUVEC5,6_ tryp_HCDorbi_4hr_F r8	155102	Intron retention event	Insertion	Yes (PB.7781.8)	https://genome.ucsc.edu/cgi-bin/hgTracks?db=hg38&lastVirtModeType=customUrl&lastVirtModeExtraState=multiRegionsBedUrl%3D%2Fuserdata%2Fsessions%2Frr%2F13%2Fmm5db%2F0c7bcefc%2Fhgt%2FcustRgn_genome_1e56d_a22290.bed+0&virtModeType=customUrl&virtMod	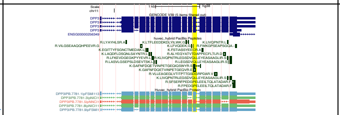	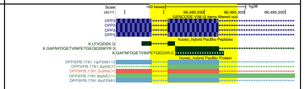
EPB41L3	PB.11510.2 5	QEDAPMIEPL VPEEVSSQK	6.390643	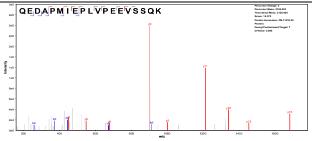	Minimum 9 amino acids, Score>5, delta mass<5ppm, PEP<0.005, good b and y ion series, extra peaks do not exceed 25% above 10% relative intensity; MS2 TIC approx 37% (rough minimum is 20%)	Yes	210827_HUVEC5,6_ tryp_HCDorbi_4hr_F r6	151428	Alternative donor (5' splice site)	Deletion	Yes (PB.11510.2 5)		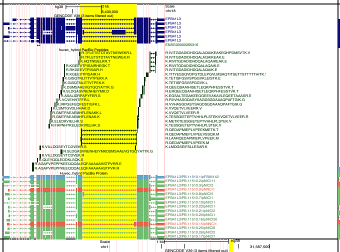	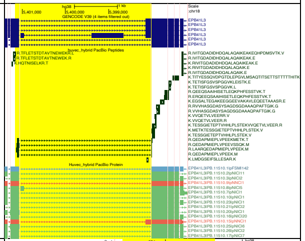
TINAGL1	PB.306.19|P B.306.2	GATSASCAAS MSATSR		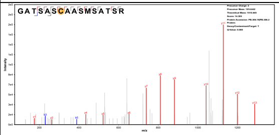	Minimum 9 amino acids, Score>5, delta mass<5ppm, PEP<0.005, good b and y ion series, extra peaks do not exceed 25% above 10% relative intensity; MS2 TIC approx 30% (rough minimum is 20%)	Yes	210827_HUVEC5,6_ tryp_HCDorbi_4hr_F r9	32118	Frameshift	Change of frame	No (PB.306.19, PB.306.2)	https://genome.ucsc.edu/cgi-bin/hgTracks?db=hg38&lastVirtModeType=customUrl&lastVirtModeExtraState=multiRegionsBedUrl%3D%2Fuserdata%2Fsessions%2Frr%2F13%2Fmm5db%2F0c7bcefc%2Fhgt%2FcustRgn_genome_1e56d_a22290.bed+0&virtModeType=customUrl&virtMode=1&multiRegionsBedUrl=%2Fuserdata%2Fsessions%2Frr%2F1	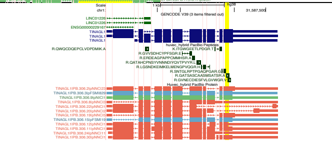	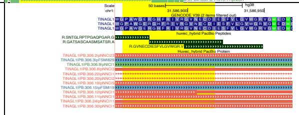

TINAGL1	PB.306.19|PB.306.2	GATSASCAASMSATSR		same sequence as above			210827_HUVEC5,6_tryp_HCDorbi_4hr_Fr10	109263						
CHID1	PB.7408.15|PB.7408.19	DAREPVVGASVHGAS		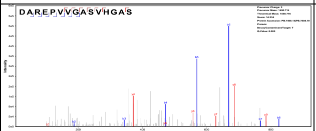	Minimum 9 amino acids, Score>5, delta mass<5ppm, PEP<0.005, good b and y ion series, extra peaks do not exceed 23% above 10% relative intensity; MS2 TIC approx 30% (rough minimum is 20%)	Yes	210827_HUVEC5,6_tryp_HCDorbi_4hr_Fr4	40229	Intron retention event	Insertion	No (PB.7408.15, PB.7408.19)	https://genome.ucsc.edu/cgi-bin/hgTracks?db=hg38&lastVirtModeType=customUrl&lastVirtModeExtraState=multiRegionsBedUrl%3D%2Fuserdata%2Fsessions%2Frr%2F13%2Fmm5db%2F0c7bcefc%2Fhgt%2FcustRgn_genome_1e56d_a22290.bed+0&virtModeType=customUrl&virtMode=1&multiRegionsBedUrl=%2Fuserdata%2Fsessions%2Frr%2F13%2Fmm5db%2F0c7bcefc%2Fhgt%2FcustRgn_genome_1e56d	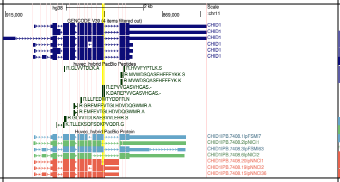	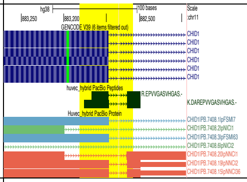
GRIPAP1	PB.13644.7	LTATSASQIQTAK	4.135122				210827_HUVEC5,6_tryp_HCDorbi_4hr_Fr10	37590						
ELAVL1	PB.11846.6	VLVDQTTGSSEPITVK	2.631441				210827_HUVEC5,6_tryp_HCDorbi_4hr_Fr9	76688						
BCAP29	PB.5544.41|PB.5544.6	SGGGFSAIDNPR					210827_HUVEC5,6_tryp_HCDorbi_4hr_Fr7	50839						
TPX2	PB.12746.8	SVAVQEPFQLATEKR	7.894324				210827_HUVEC5,6_tryp_HCDorbi_4hr_Fr11	94947						
EWSR1	PB.13268.8	AAVEWFDGPGGPGGPGGPMGR	10.14985				210827_HUVEC5,6_tryp_HCDorbi_4hr_Fr12	154876						
HNRNPA3	PB.2087.35|PB.2087.7	GSYGGGDGGYNGFGGDGNYGGGGNYNDFGNYSGQQQSNYGPMK					210827_HUVEC5,6_tryp_HCDorbi_4hr_Fr10	148370						
TINAGL1	PB.306.19|PB.306.2	SNTGLRPTPGAQPGAR					210827_HUVEC5,6_tryp_HCDorbi_4hr_Fr10	24350						
FLI1	PB.8115.18	LSVKEDPYQILGPTSSR	5.638803				210827_HUVEC5,6_tryp_HCDorbi_4hr_Fr13	108391						
MCAM	PB.8064.40	RAPGDQAGFHHPGAPLK	1.503681				210827_HUVEC5,6_tryp_HCDorbi_4hr_Fr11	32063						
ACROH2A	PB.4095.17	SLFLGQKPPQ	0.75184				210827_HUVEC5,6_tryp_HCDorbi_4hr_Fr15	71361						
NCOA5	PB.12877.2|PB.12877.7	SSTDSLPGELR					210827_HUVEC5,6_tryp_HCDorbi_4hr_Fr4	70139						
MFF	PB.2279.19	FQAPISAPEYTPVLR	1.879601				210827_HUVEC5,6_tryp_HCDorbi_4hr_Fr14	140104						
XRCC6	PB.13420.6	NLEALALDLMEPEQAVDLTYNEGSGSK	2.255521				210827_HUVEC5,6_tryp_HCDorbi_4hr_Fr11	242933						
CHID1	PB.7408.15|PB.7408.19	EPVVGASVHGAS					210827_HUVEC5,6_tryp_HCDorbi_4hr_Fr4	35986						
EDC4	PB.10482.10|PB.10482.9	DGGSILGLGR					210827_HUVEC5,6_tryp_HCDorbi_4hr_Fr12	95904						
IARS1	PB.6502.7	EIVDQITEDIPVK	0.75184				210827_HUVEC5,6_tryp_HCDorbi_4hr_Fr6	157285						
GSN	PB.6628.19|PB.6628.23	LCPVSSQPNSMVVEHPEFLK					210827_HUVEC5,6_tryp_HCDorbi_4hr_Fr13	121441						
MAP4	PB.2568.30	TKPAPTTIGGLNK	1.503681				210827_HUVEC5,6_tryp_HCDorbi_4hr_Fr14	27639						
PDLIM4	PB.4059.7	YLQGMLEAGEGGK	1.127761				210827_HUVEC5,6_tryp_HCDorbi_4hr_Fr12	91619						
NR2C2	PB.2424.3	VGTQQVAEVTR	0.75184				210827_HUVEC5,6_tryp_HCDorbi_4hr_Fr6	41243						
MAP4	PB.2568.18	SLGMASGSGSPTEFLEEK	0.75184				210827_HUVEC5,6_tryp_HCDorbi_4hr_Fr9	123944						
MAP4	PB.2568.18	SLGMASGSGSPTEFLEEK	0.75184				210827_HUVEC5,6_tryp_HCDorbi_4hr_Fr11	181881						

SREBF1	PB.10847.6	LLCAFAVEGPR	0.75184				210827_HUVEC5,6_tryp_HCDorbi_4hr_Fr14	93055						
ITM2B	PB.8961.3	TAAAVRPEVAGAGLAPR	0.75184				210827_HUVEC5,6_tryp_HCDorbi_4hr_Fr11	60015						
EFHD2	PB.137.2	EVQAINVSSR	6.766564				210827_HUVEC5,6_tryp_HCDorbi_4hr_Fr7	35451						
YEATS2	PB.3076.14	AGFPASTEAER	0.75184				210827_HUVEC5,6_tryp_HCDorbi_4hr_Fr4	42500						
UBAP2L	PB.927.12	QQEEQDILNFVDDQLGE	1.879601				210827_HUVEC5,6_tryp_HCDorbi_4hr_Fr6	243852						
KDELR2	PB.5090.2	NPPTSTSHVAGTTGISGK	1.879601				210827_HUVEC5,6_tryp_HCDorbi_4hr_Fr8	29808						
GRIPAP1	PB.13644.7	SRLTATSASQIQTAK	4.135122				210827_HUVEC5,6_tryp_HCDorbi_4hr_Fr15	30790						
EDC4	PB.10482.10|PB.10482.9	ILETGSTTWHR					210827_HUVEC5,6_tryp_HCDorbi_4hr_Fr13	48822						
GAK	PB.3183.4|PB.3183.5	EEQQDILSKFEEKEAETGAENASSK					210827_HUVEC5,6_tryp_HCDorbi_4hr_Fr4	132985						
NR2C2	PB.2424.3	TQTSRPESPGMTSPSPR	0.75184				210827_HUVEC5,6_tryp_HCDorbi_4hr_Fr6	33528						
NR2C2	PB.2424.3	TQTSRPESPGMTSPSPR	0.75184				210827_HUVEC5,6_tryp_HCDorbi_4hr_Fr9	34724						
PXDN	PB.1416.36|PB.1416.42	YFGSPALPQFTVTPQDR					210827_HUVEC5,6_tryp_HCDorbi_4hr_Fr18	149239						
CNPY3	PB.4643.4	SISDPPDQMTYLPSSSESLPIGGELSSSSSCLGR	3.383282				210827_HUVEC5,6_tryp_HCDorbi_4hr_Fr11	202088						
PDIA5	PB.2842.8	TVSGGEASAQSSLSADGQGNFH	9.398005				210827_HUVEC5,6_tryp_HCDorbi_4hr_Fr5	71748						
PDCD6IP	PB.2480.13	TMPVLPNAHAHGL	1.503681				210827_HUVEC5,6_tryp_HCDorbi_4hr_Fr13	78462						
POLE3	PB.6603.3	IIKEAVSSSGGADER	7.142484				210827_HUVEC5,6_tryp_HCDorbi_4hr_Fr9	30071						
SDCCAG8	PB.1381.15|PB.1381.8	QEETLREQTLLDASEKLK					210827_HUVEC5,6_tryp_HCDorbi_4hr_Fr5	82884						
TCIRG1	PB.7820.23	GPAAAATAEPGAAGGPR	3.007362				210827_HUVEC5,6_tryp_HCDorbi_4hr_Fr6	39730						
TINAGL1	PB.306.12	LGSNDKEIMKELMENGPVQGR	1.127761				210827_HUVEC5,6_tryp_HCDorbi_4hr_Fr9	140297						
TINAGL1	PB.306.12	LGSNDKEIMKELMENGPVQGR	1.127761				210827_HUVEC5,6_tryp_HCDorbi_4hr_Fr9	140115						
TINAGL1	PB.306.12	LGSNDKEIMKELMENGPVQGR	1.127761				210827_HUVEC5,6_tryp_HCDorbi_4hr_Fr10	132634						
RASIP1	PB.12413.27	KSPMEEEIGNSSLYSK	0.75184				210827_HUVEC5,6_tryp_HCDorbi_4hr_Fr5	120513						
RASIP1	PB.12413.27	KSPMEEEIGNSSLYSK	0.75184				210827_HUVEC5,6_tryp_HCDorbi_4hr_Fr14	164276						
EIF3C	PB.10293.6	MIINEELMASLDQPTQTVVMHRTEPGQPGGEQR	1.503681				210827_HUVEC5,6_tryp_HCDorbi_4hr_Fr9	151197						
DKC1	PB.13939.6	AGLESGAEPGDGVCGNTFR	0.75184				210827_HUVEC5,6_tryp_HCDorbi_4hr_Fr6	93928						
CRACR2A	PB.8149.1	SSQSEEEEEVFGIPR	4.886963				210827_HUVEC5,6_tryp_HCDorbi_4hr_Fr4	135604						
EIF4G1	PB.3084.13	APQSTGPPPAPSPGLPQHFYPSR	0.75184				210827_HUVEC5,6_tryp_HCDorbi_4hr_Fr14	93359						
PRPF31	PB.12514.10	KQANRMSFGEVR	0.75184				210827_HUVEC5,6_tryp_HCDorbi_4hr_Fr5	49333						
PRPF31	PB.12514.10	KQANRMSFGEVR	0.75184				210827_HUVEC5,6_tryp_HCDorbi_4hr_Fr17	60035						
TPM1	PB.9793.11	QAEADVASLNRR	1.127761				210827_HUVEC5,6_tryp_HCDorbi_4hr_Fr6	36261						
SFPQ	PB.348.12	STKEILIFTT	4.886963				210827_HUVEC5,6_tryp_HCDorbi_4hr_Fr15	111484						
LOXL2	PB.5833.22	HDIDCQWVDITDVPPGDYLFQVVPSAK	25.93849				210827_HUVEC5,6_tryp_HCDorbi_4hr_Fr14	223538						

LRCH3	PB.3162.17	IGVPQELLK	0.75184				210827_HUVEC5,6_tryp_HCDorbi_4hr_Fr15	107154						
SMARCC2	PB.8453.16	EGERAGGEEVR	0.75184				210827_HUVEC5,6_tryp_HCDorbi_4hr_Fr9	131372						
SPOUT1	PB.6707.6	LNQQQHPDSVTPPDCK	1.127761				210827_HUVEC5,6_tryp_HCDorbi_4hr_Fr4	32468						
ZMYND8	PB.12885.11	TFSMADPGSAER	4.511042				210827_HUVEC5,6_tryp_HCDorbi_4hr_Fr7	63314						
GLA	PB.13773.3	NFEAMVAEPK	4.886963				210827_HUVEC5,6_tryp_HCDorbi_4hr_Fr8	82400						
SLC25A29	PB.9503.16	GSGVPPMQLSPGGPGR	3.759202				210827_HUVEC5,6_tryp_HCDorbi_4hr_Fr11	28193						
FTSJ1	PB.13630.7	CDSQPALTPSPCAFF	1.127761				210827_HUVEC5,6_tryp_HCDorbi_4hr_Fr12	210744						
SCRN1	PB.5171.16	SQAEEHHAGAGEAR	0.75184				210827_HUVEC5,6_tryp_HCDorbi_4hr_Fr7	34226						
PRXL2B	PB.54.12	GVSSQPWEGWGLSK	0.75184				210827_HUVEC5,6_tryp_HCDorbi_4hr_Fr11	137263						
UAP1L1	PB.6811.13	AVTRCAAAGGCGLPAR	0.75184				210827_HUVEC5,6_tryp_HCDorbi_4hr_Fr7	145605						
ZEB1	PB.6948.22	QANPRRNNAGK	0.75184				210827_HUVEC5,6_tryp_HCDorbi_4hr_Fr12	174748						
LAMA4	PB.4847.99	TLTINAFQHQQCDGTPR	6.766564				210827_HUVEC5,6_tryp_HCDorbi_4hr_Fr13	78624						
U2SURP	PB.2954.16	LREIEEKEK	0.75184				210827_HUVEC5,6_tryp_HCDorbi_4hr_Fr7	54435						
RAN	PB.8811.11	RNAAMAAQGEPQVQFK	3.007362				210827_HUVEC5,6_tryp_HCDorbi_4hr_Fr6	86809						
TM9SF4	PB.12751.12	GLGWGGMKLDGEGR	1.127761				210827_HUVEC5,6_tryp_HCDorbi_4hr_Fr10	157495						
TM9SF4	PB.12751.12	GLGWGGMKLDGEGR	1.127761				210827_HUVEC5,6_tryp_HCDorbi_4hr_Fr6	121642						
TXNRD1	PB.8628.12	QLDSTIGIHPVCAEGSCHPGWSAVV	4.511042				210827_HUVEC5,6_tryp_HCDorbi_4hr_Fr12	155884						
PRKAR2A	PB.2585.28	KIWHHLQFHRITVK	0.75184				210827_HUVEC5,6_tryp_HCDorbi_4hr_Fr14	45225						
MAPRE1	PB.12763.2	IVDILYATDVCV	4.135122				210827_HUVEC5,6_tryp_HCDorbi_4hr_Fr15	202173						
GSPT1	PB.10200.17	GPAAPPPPVGGAANNHGAGSGAGGRADAGK	0.75184				210827_HUVEC5,6_tryp_HCDorbi_4hr_Fr7	49405						
TSN	PB.1936.15	ELSQIGR	1.127761				210827_HUVEC5,6_tryp_HCDorbi_4hr_Fr11	79552						
ARHGDIA	PB.11451.3	CSQGAAQGWGFAQGCWAVIEGEVPPTGDLESFKK	6.766564				210827_HUVEC5,6_tryp_HCDorbi_4hr_Fr14	216610						
GART	PB.13065.1	VQGILLLQPPE	3.759202				210827_HUVEC5,6_tryp_HCDorbi_4hr_Fr16	163319						
